# Internal and External Validation of a multivariable Model to Define Hospital-Acquired Pneumonia After Esophagectomy

**DOI:** 10.1007/s11605-016-3083-5

**Published:** 2016-02-16

**Authors:** Teus J. Weijs, Maarten F. J. Seesing, Peter S. N. van Rossum, Marijn Koëter, Pieter C. van der Sluis, Misha D. P. Luyer, Jelle P. Ruurda, Grard A. P. Nieuwenhuijzen, Richard van Hillegersberg

**Affiliations:** Department of Surgery, Catharina Hospital Eindhoven, Eindhoven, The Netherlands; Department of Surgery, University Medical Center Utrecht, Heidelberglaan 100, 3508 GA Utrecht, The Netherlands; Department of Surgical Oncology, UMC Utrecht, Heidelberglaan 100, 3508 GA Utrecht, The Netherlands; Department of Radiotherapy, University Medical Center Utrecht, Utrecht, The Netherlands

**Keywords:** Esophageal carcinoma, Esophagectomy, Pneumonia

## Abstract

**Background:**

Pneumonia is an important complication following esophagectomy; however, a wide range of pneumonia incidence is reported. The lack of one generally accepted definition prevents valid inter-study comparisons. We aimed to simplify and validate an existing scoring model to define pneumonia following esophagectomy.

**Patients and methods:**

The Utrecht Pneumonia Score, comprising of pulmonary radiography findings, leucocyte count, and temperature, was simplified and internally validated using bootstrapping in the dataset (*n* = 185) in which it was developed. Subsequently, the intercept and (shrunk) coefficients of the developed multivariable logistic regression model were applied to an external dataset (*n* = 201)

**Results:**

In the revised Uniform Pneumonia Score, points are assigned based on the temperature, the leucocyte, and the findings of pulmonary radiography. The model discrimination was excellent in the internal validation set and in the external validation set (C-statistics 0.93 and 0.91, respectively); furthermore, the model calibrated well in both cohorts.

**Conclusion:**

The revised Uniform Pneumonia Score (rUPS) can serve as a means to define post-esophagectomy pneumonia. Utilization of a uniform definition for pneumonia will improve inter-study comparability and improve the evaluations of new therapeutic strategies to reduce the pneumonia incidence.

## Background

Each year, esophageal cancer is diagnosed in 460,000 people worldwide.^1^ Esophagectomy is the corner stone of treatment for resectable esophageal cancer. However, this complex procedure^2^ is associated with a high rate of complications that need an invasive re-intervention (27–38 %)^3^^–^6 and a high 90-day mortality rate (approximately 9 %).^7^ Pulmonary complications are most frequently observed and significantly increase the intensive care unit readmission rate, the length of hospital stay, and the mortality rate.^8^^,^^9^ This stresses the need for strategies to reduce these complications. However, research in pneumonia following esophagectomy—which is the most common postoperative pulmonary complication—is frustrated by the lack of a widely accepted definition that is easy to apply.

The reporting of pneumonia was investigated in a recent systematic review of prospective studies conducted between 2004 and 2009 including more than 50 patients undergoing esophagectomy.^10^ Pneumonia rates were reported by 56 studies but defined by 18 studies only, using 16 different definitions. The variation across reported pneumonia rates was large, ranging from 2 to 39 %.^10^ Since the range of pneumonia incidence is so strongly determined by definition, valid inter-study of even within-study comparisons cannot be made.

Recently, an objective scoring system to define pneumonia at the ward following esophagectomy was developed (Table 1).^8^ In the Utrecht Pneumonia Score, pneumonia is defined based on temperature, leucocyte count, and pulmonary radiography findings. The aim of this study was to internally and externally validate the Utrecht Pneumonia Score as definition for pneumonia at the ward in patients following esophagectomy.

**Table 1:** Original and revised Uniform Pneumonia Score, a definition for hospital-acquired pneumonia after esophagectomy

	Utrecht Pneumonia Score		Revised Uniform Pneumonia Score	
Diagnostic determinant	Range	Score	Range	Score
Temperature [°C]	≥36.1 and ≤38.4	0	≥36.1 and ≤38.4	0
	≥38.5 and ≤38.9	1	≤36.0 and ≥38.5	1
	≥39.0 and ≤36.0	2		
Leukocyte count [×10^9^/L]	≥4.0 and ≤11.0	0	≥4.0 and ≤11.0	0
	<4.0 or >11.0	1	<4.0 or >11.0	1
Pulmonary radiography	No infiltrate	0	No infiltrate	0
	Diffused (or patchy) infiltrate	1	Diffused (or patchy) infiltrate	1
	Well-circumscribed infiltrate	2	Well-circumscribed infiltrate	2

A sum score of 2 points or higher, of which at least 1 point is assigned due to infiltrative findings on pulmonary radiography, indicates treatment of pneumonia

## Patients and Methods

Approval was obtained from the local medical ethical committee, and the need for informed consent was waived for this study. Analysis and reporting were performed in accordance with the TRIPOD statement.^11^ For the internal validation, the original development set of the UPS was used,^8^ consisting of a consecutive cohort of patients that underwent an esophagectomy with gastric conduit reconstruction in the University Medical Center Utrecht between October 2003 and March 2011. For the external validation, a cohort of all consecutive patients that underwent an esophagectomy in the Catharina Hospital Eindhoven between January 2008 and March 2014 was used. Data for both cohorts were extracted from prospectively acquired databases which contain patient characteristics and intraoperative and postoperative data. Model variables had to be collected retrospectively. Exclusion criteria were presence of pneumonia at the time of surgery and death before model variables could be measured. In addition, complete case analysis was performed by excluding patients with missing values for model variables, as the amount of missing data was low.

### Surgical Procedure and Postoperative Care

Patients underwent either open or minimally invasive transthoracic or transhiatal esophagectomy with or without robot assistance. Epidural analgesia was administered routinely during and following surgery. A gastric conduit reconstruction was performed with either a cervical or an intrathoracic anastomosis. Bilateral chest tubes were placed at the end of transthoracic surgery. In the cohort that was used to develop the UPS, a feeding jejunostomy was created in all patients to bridge the nil-by-mouth period in the first 5 to 7 days after esophagectomy. In the validation cohort, the same postoperative nutritional regimen was used except for a subset in which the feasibility and safety of direct oral intake following esophagectomy were investigated.^12^ All patients were transferred to the intensive care unit postoperatively. When patients were respiratory and hemodynamically stable without support or intensive monitoring, they were transferred to the surgical ward.

### Outcome Definition

Currently, there is no established well-defined gold standard for diagnosing pneumonia after esophagectomy.^10^ Therefore, pneumonia was defined as the clinical decision to treat suspected pneumonia, similarly to van der Sluis et al..^8^ Treatment for pneumonia, unless contra-indicated, was primarily by intravenous cefuroxime or ceftriaxone.

### Predictors

In accordance with van der Sluis et al.,^8^ data for the following three diagnostic determinants of interest were retrospectively collected from the patients’ charts of the medium care unit or hospital ward stay: temperature (°C), leukocyte count (×10^9^/L), and pulmonary radiography findings. Pulmonary radiography findings could include both chest X-rays and/or CT scans depending on the availability, preferring CT over conventional X-rays when both were available. In patients not treated for pneumonia, the temperature, leucocyte count, and pulmonary radiography were collected on the fourth day at the hospital ward to ensure a sufficient time from ICU discharge. In this study, the original UPS was simplified: the revised Uniform Pneumonia Score (rUPS). The same cutoff values as used by van der Sluis et al. were applied for pulmonary radiography and leucocyte count (Table 1). In the original model, 0, 1, or 2 points could be attributed for temperature. Since the cutoff value for pneumonia is 2 points, with at least 1 point assigned based on pulmonary radiography, it does not matter if 1 or 2 points are assigned for temperature. Thus, in the rUPS, 0 or 1 point could be assigned for temperature (≥36.1 and ≤38.4 °C = 0 points and ≤36.0 or ≥38.5 °C = 1 point).

### Statistical Analysis

Data were analyzed using SPSS for windows, version 22.0 (IBM Corp., Armonk, New York) and R 3.1.2 open-source software (http://www.R-project.org). All continuous data were presented as median (25th percentile–75th percentile), and all categorical data were presented as number (percentage).

Patient and treatment-related characteristics as well as postoperative outcomes besides pneumonia were compared between the development cohort and external cohort to gain insight in potential differences. Continuous data were compared using the Mann-Whitney *U* test, and categorical data were compared using the chi-squared test. Odds ratios (ORs) along with 95 % confidence intervals (CIs) for each variable of the revised Uniform Pneumonia Score were calculated in the development set.

First, the model performance of the rUPS was assessed for discriminatory ability and calibration in the development set. The ability to distinguish a patient with the outcome from a patient without the outcome is indicated by discrimination, which was assessed by using the concordance (C) statistic. Calibration refers to the agreement between predicted probability of pneumonia by the model and the observed probability and was assessed by visual inspection of calibration plots. Second, internal validation of the rUPS was performed by applying bootstrap re-sampling with 200 repetitions in the development set. The C-statistics of the original model in the 200 bootstrap samples were averaged, and the optimism was indicated by the differences between this average C-statistic and the original C-statistic.^13^ As such, bootstrapping allowed for adjustment of the model performance for optimism caused by model overfitting and additionally provided a uniform shrinkage factor that was used to adjust the original model coefficients. Third, the same three variables of the rUPS that were included in the internal validation were used for external validation, in which the original intercept and shrunk coefficients after internal validation of the model were applied. Then, external discriminatory ability and calibration were determined.

## Results

### Inclusion

**Fig. 1:**
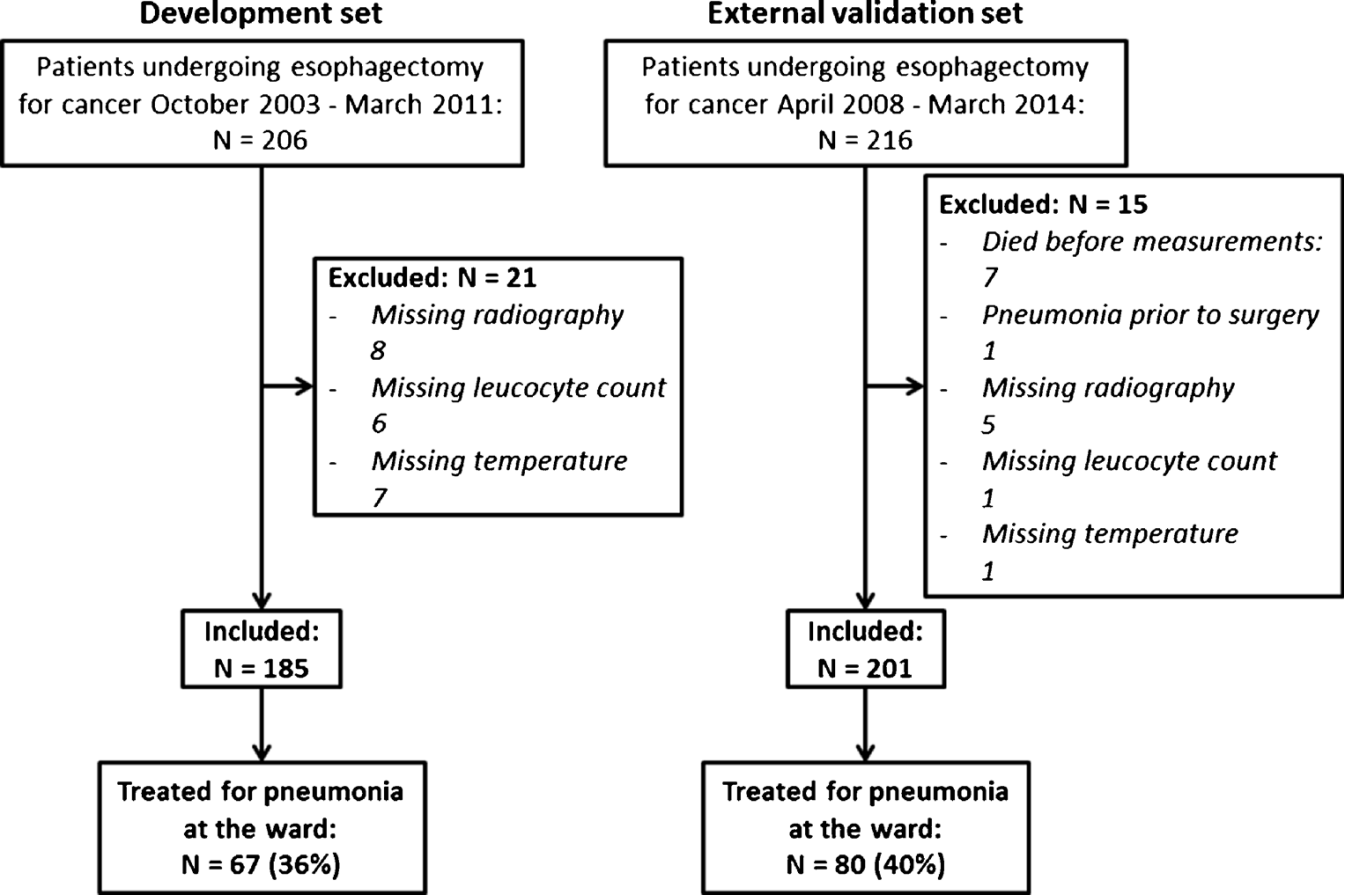
Patient flow

The process of patient selection is shown in Fig. 1. For the development dataset, 185 patients were included, of who 67 patients (36 %) were treated for pneumonia at the ward. The external validation dataset finally consisted of 201 patients of whom 80 patients (40 %) were treated for pneumonia at the ward.

### Patient Characteristics

The patient and treatment-related characteristics of the development and validation datasets are presented in Tables 2 and 3. Significant differences between both cohorts included the higher rate of ASA 1 patients, T3–T4 tumors, N2–N3 tumors, cervical anastomosis, recurrent nerve injury, lymph nodes resected, and a longer hospital admission time among patients in the development set. In the external cohort, the rate of alcohol use, neoadjuvant chemoradiation, intrathoracic anastomosis, transhiatal surgery, and direct start of oral intake was significantly higher compared to the development cohort.

**Table 2:** Patient characteristics

Variable	Internal validation study (*n* = 185)	External validation study (*n* = 201)	*p* Value
Gender			
Male	141 (76)	168 (80)	0.356^a^
Female	44 (24)	40 (19)	
Age	64 (58–71)	64 (57–70)	0.533^b^
Tobacco user	95 (51)	111 (55)^c^	0.474^a^
Alcohol user	102 (55)	132 (66)^c^	0.025^a^
Comorbidity			
Cardial	37 (20)	42 (20)	0.327^a^
Diabetes	26 (14)	23 (11)	0.441^a^
COPD	23 (12)	32 (15)	0.606^a^
ASA score			
I	47 (25)	24 (12)	0.007^b^
II	106 (57)	144 (70)	
III	31 (17)	39 (19)	
IV	1 (<1)	1 (<1)	
Body mass index (kg/m^2^)	25 (23–28)	26 (23–28)^c^	0.500^b^
Tumor infiltration		^c^	
Complete pathologic response	9 (5)	45 (23)	0.000^a^
I	36 (19)	44 (22)	
II	16 (9)	43 (21)	
III	118 (64)	69 (34)	
IV	6 (3)	0	
Lymph node metastasis			
N0	72 (39)	113 (54)	0.000^a^
N1	51 (28)	52 (25)	
N2	41 (22)	22 (11)	
N3	21 (11)	3 (5)	
Neoadjuvant therapy			
None	114 (62)	20 (10)	0.000^a^
Chemotherapy	63 (34)	20 (10)	
Chemoradiotherapy	8 (4)	167 (80)	
Radiotherapy	0	1 (<1)	

Table showing the baseline data. For continuous variables, data shown represent median (interquartile range); all other data are presented as numbers (percentages)

*n* number, *ASA score* American Society of Anesthesiologist score

^a^Two-sided chi-squared test

^b^Mann-Whitney *U* test

^c^Missing, tobacco user *n* = 1, alcohol user *n* = 2, body mass index *n* = 4, depth of tumor infiltration *n* = 1

**Table 3:** Surgical characteristics and clinical outcome

	Internal validation study (*n* = 185)	External validation study (*n* = 201)	*p* Value
Surgical approach			
Open transhiatal	29 (16)	42 (20)	0.000^a^
Open transthoracic	11 (6)	1 (1)	
Minimally invasive, transhiatal	22 (12)	61 (29)	
Minimally invasive, transthoracic	123 (67)	104 (50)	
Level of anastomosis			
Intrathoracic	1 (1)	63 (30)	0.000^a^
Cervical	184 (99)	144 (69)	
Lymph nodes resected	19 (13–27)	15 (9–23)	0.000^b^
Early start of oral intake	0 (0)	29 (17)	0.000^a^
Clinical outcome			
Anastomotic leakage	41 (22)	60 (29)	0.132^a^
ARDS	3 (2)	2 (1)	0.587^a^
Recurrent laryngeal nerve injury	18 (10)	6 (3)	0.006^a^
Cardiac arrythmia	23 (12)	34 (16)	0.215^a^
Chyle leakage	26 (14)	25 (12)	0.537^a^
Intensive care unit re-admission	34 (18)	46 (22)	0.275^a^
Hospital admission time	17 (13–25)	13 (10–22)	0.000^b^

Table showing the characteristics of the surgery performed and clinical outcome. For continuous variables, data shown represent median (25th percentile–75th percentile); all other data are numbers (percentages). There were no missing data

^a^Two-sided chi-squared test

^b^Mann-Whitney *U* test

### Multivariable Regression Model

The multivariable logistic regression model of the simplified rUPS in the prediction of treatment for pneumonia is presented in Table 4. A temperature score of 1 was significantly and independently associated with a higher chance of treatment for pneumonia (OR 12.0, *p* = 0.001). A score of 1 for leucocyte count was significantly and independently associated with a higher chance of treatment for pneumonia (OR 6.0, *p* = 0.006). Pulmonary radiography findings were significantly and independently associated with an increased chance for pneumonia treatment (1 point: OR 37.4, *p* = 0.000; 2 points: OR indefinite).

**Table 4:** The revised Uniform Pneumonia Score

	Treated (*n* = 67)	Not treated (*n* = 118)	Odd ratios (95 % confidence interval)	*p* Value
Temperature				
≥36.1 and ≤38.4	45 (29)	112 (71)	Reference	
≤36.0 and ≥38.5	22 (79)	6 (21)	12.0 (2.8–51.1)	0.001
Leucocytes				
≥4.0 and ≤11.0	9 (11)	75 (89)	Reference	
<4.0 and >11.0	58 (57)	43 (43)	6.0 (1.7–21.6)	0.006
Pulmonary radiography				
No infiltrate	13 (10)	113 (90)	Reference	
Diffuse or patchy infiltrate	31 (86)	5 (14)	37.4 (11.0–127.4)	0.000
Well-circumscribed infiltrate	23 (100)	0 (0)	Indefinite	0.000

Table showing independent odds ratios of the components of the revised Uniform Pneumonia Score, calculated by logistic regression. Values presented are numbers (percentages) and odds ratios (95 % confidence interval)

### Internal Validation

**Fig. 2:**
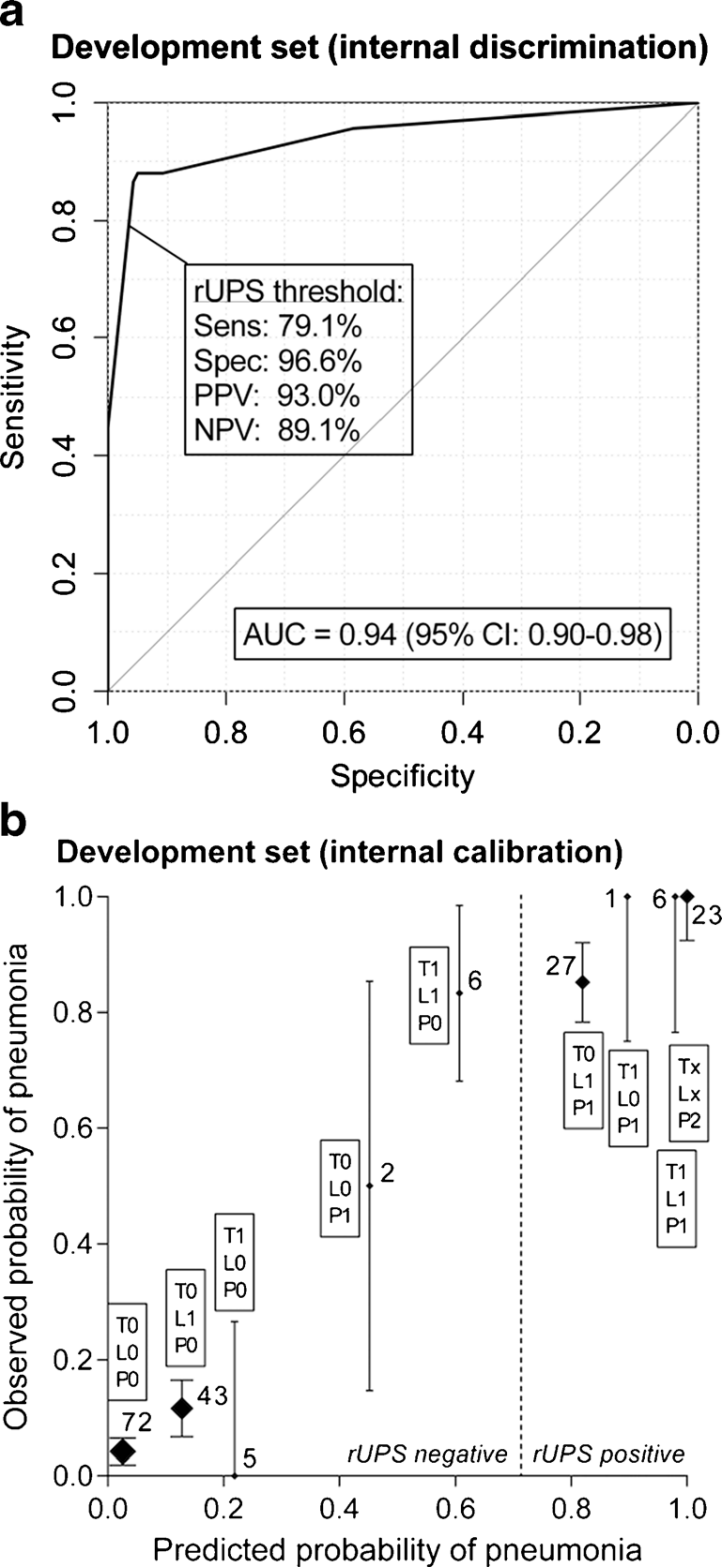
Internal validation: **a** discrimination and **b** calibration. The predicted probability of pneumonia is calculated by the sum of the predictive values of every independent variable multiplied by presence of every variable. The observed probability of pneumonia is the percentage of patients treated for pneumonia at the ward at any given point on the *x*-axis. *Sens* sensitivity, *Spec* specificity, *PPV* positive predictive value, *NPV* negative predictive value, *T* temperature, *L* leukocytes, *P* pulmonary radiography

The rUPS shows an excellent discriminatory ability and calibration in the development set (Fig. 2a–b), with an apparent C-statistic of 0.94. Internal validation by bootstrapping resulted in an adjusted C-statistic of 0.94, representing hardly any optimism (i.e., 0.004) due to overfitting. Using the predefined cutoff value (at least 2 points of which at last 1 has to be assigned for pulmonary radiography), the sensitivity was 79 % and the specificity was 97 %. Slight miscalibration was observed in the group of patients (*n* = 6) with 2 points assigned, based on leucocyte count and temperature and the group of patients (*n* = 2) with 1 point only, assigned for radiography. The observed probability of pneumonia treatment was relatively high in these two groups, while they were not classified as pneumonia by the revised UPS. Only one patient of the three patients that had ARDS had this at the moment pneumonia treatment was initiated, and the pneumonia score was measured.

### External Validation

**Fig. 3:**
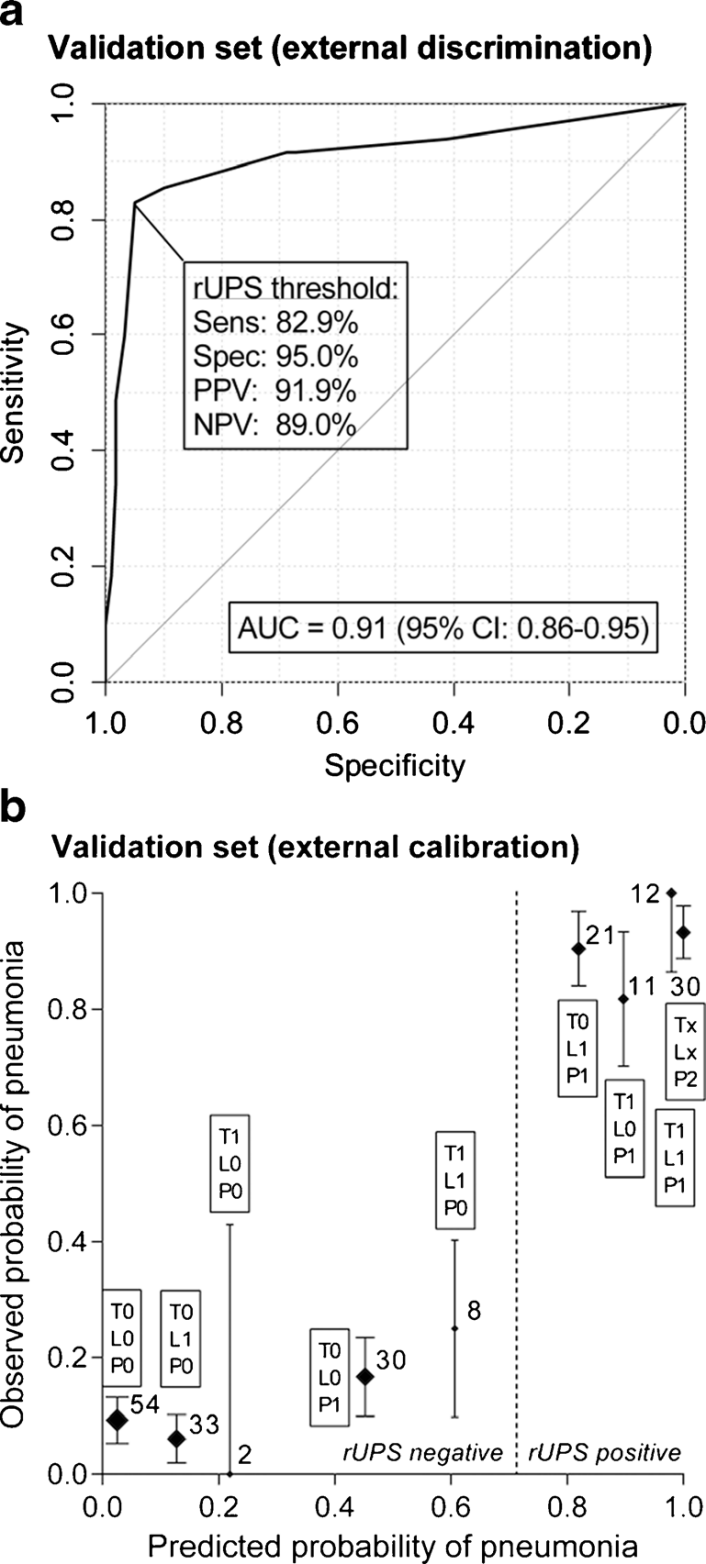
External validation: **a** discrimination and **b** calibration. The predicted probability of pneumonia is calculated by the sum of the predictive values of every independent variable multiplied by presence of every variable. The observed probability of pneumonia is the percentage of patients treated for pneumonia at the ward at any given point on the *x*-axis. *Sens* sensitivity, *Spec* specificity, *PPV* positive predictive value, *NPV* negative predictive value, *T* temperature, *L* leukocytes, *P* pulmonary radiography

The rUPS shows an excellent discriminatory ability (C-statistic of 0.91) and calibration in the validation set (Fig. 3a–b). The sensitivity was 83 %, and the specificity was 95 % for the predefined cutoff value. The calibration plot showed a low observed probability of pneumonia treatment in all patient groups that were classified as not having pneumonia and a high probability of pneumonia treatment in all patient groups with a high predicted probability of pneumonia. None of the two patients with ARDS had this at the moment that the pneumonia score was measured.

## Discussion

The necessity for a uniform registration and definition of pneumonia following esophagectomy has become apparent by the article of Blencowe et al..^10^ Now, we show that the revised Uniform Pneumonia Score discriminates pneumonia from no pneumonia correctly in more than 90 % of cases in internal and external validation sets with excellent calibration. This score consists of easy to measure variables: temperature, leucocyte count, and pulmonary radiography findings.

The main finding of the study by Blencowe et al. was that the large range in pneumonia definitions (16 different definitions) used by studies resulted in an equally large range of reported pneumonia rates (2–39 %).^10^ Among others, this study resulted in the initiative to standardize outcome reporting following esophagectomy, recently published as the International Consensus on Standardization of Data Collection for Complications Associated with Esophagectomy.^14^ However, for reasons not stated, no consensus statement was made regarding pneumonia. For the definition of pneumonia, the reader was referred to the guidelines of the American Thoracic Society and Infectious Diseases Society of America.^15^ In these guidelines, focusing on ventilator-associated pneumonia (VAP), the main reference is the clinical pulmonary infection score (CPIS), which has been extensively investigated for VAP.^16^^–^18 The gold standard in these studies were postmortem investigation or microbiological results of bronchoalveolar lavage fluids, both not without flaws.^16^ Also, studies show that the CPIS criteria are not applicable to specific patient groups, such as trauma patients.^19^^,^^20^ For hospital-acquired pneumonia (HAP), no scoring system was proposed nor investigated. This shows the need for the present study in which hospital-acquired pneumonia was investigated.

This study has several strengths. The model was developed and revised in a prospectively maintained database including a large cohort in relation to the number of variables studied. There was no loss to follow-up, and numbers of missing data were few. The variables used can be easily and objectively measured. In contrast to most studies, the model was internally and externally validated, confirming the good discrimination and calibration in an external cohort of patients that underwent esophagectomy. Notably, there were many significant differences between the development cohort and validation cohort, such as the application of neoadjuvant therapy. This did not impact the discriminatory abilities of the model, emphasizing that it is generally applicable.

The main limitation of the present study was the lack of a useable gold standard for the diagnosis of pneumonia. This lack of an objective definition called for a uniform and objective scoring model to improve inter-study and inter-hospital comparability, which was the main reason for this study. We chose to use the clinical decision to treat a suspected pneumonia as gold standard. Though clinical judgment is subjective and related to experience, this is the only definition in which all factors are accounted for, by the attending clinician. For research purpose however, a clearer definition is needed. Therefore, we aimed to determine what parameters accurately determined the outcome of this decision-making process. We show that our scoring model is a good reflection of the clinical practice, not only within one center but also between different centers (inter-hospital). van der Sluis et al. recently showed that the outcome of sputum culture is not relevant, probably because the sensitivity is insufficient and the results become available after the decision to treat pneumonia has been made.^8^ Bronchoalveolar lavage is considered to be too invasive to use as routine diagnostic. Another interesting method to create a gold standard would be to create a consensus statement of several experts via a Delphi procedure.^21^ It would be interesting to use this for further validation of the rUPS.

The observed miscalibration using the rUPS in the prediction of treatment for pneumonia was very low, especially in the external validation study, indicating an excellent model for determining pneumonia. The miscalibration which was observed mainly existed in patients in the development data set that scored 1 point for leucocytes and 1 point for temperature but 0 points for pulmonary radiography. Most probably, pneumonia treatment was initiated in these patients before infiltrates were visible at pulmonary radiography. Perhaps this was based on an unmeasured other factor, such as coughing, which was not included in the rUPS since it was considered to be subjective. Finally, the calculated odds ratio of 37.4 for pulmonary radiography should be considered a rather unstable estimation due to the low numbers of false-positive and false-negative pulmonary radiography test results in this cohort, as reflected by the wide 95 % CI ranging from 11.0 to 127.4. However, even the lower bound of the 95 % CI suggests that the true underlying OR in the population is likely very high. This supports the requirement of a pulmonary radiography score of ≥1 in the scoring system for pneumonia.

The incidence of pneumonia in this study as scored by the UPS is at the high end of the range of pneumonia rates published in literature (2–39 %).^10^ As shown by Blencowe et al., the most obvious underlying reason is the different definitions used in other studies.^10^ Another study by van der Sluis et al. showed that adding the requirement of a positive sputum culture decreased the pneumonia incidence from 36 to 19 %.^8^ A relevant outcome of a study in which therapies for pneumonia are investigated may consist not only of a reduced incidence but also of a reduced severity of postoperative pneumonia. Reporting of pneumonia using the rUPS does not include information on the severity of pneumonia. This can be solved by grading the severity of rUPS-defined pneumonia using the validated revised Clavien-Dindo classification of surgical complications or Accordion classification of complications.^22^^–^24

## Conclusion

The rUPS is the first internally and externally validated method that accurately predicts treatment for pneumonia following esophagectomy. Because this score proves to represent the decision-making process of clinicians to treat for pneumonia on an inter-hospital level, the rUPS can serve as a means to define post-esophagectomy pneumonia in research. Future studies and audits reporting postoperative outcomes of esophagectomy are encouraged to provide pneumonia incidences as defined by this score to improve inter-study and inter-hospital comparability.
